# Fibroblast Viability Through Mechanical and Chemical Root Surface Modifications in Periodontal Healing: An In Vitro Comparative Study

**DOI:** 10.7759/cureus.50381

**Published:** 2023-12-12

**Authors:** Baher Felemban

**Affiliations:** 1 Department of Basic and Clinical Oral Sciences, Division of Periodontology, Faculty of Dental Medicine, Umm Al Qura University, Makkah, SAU

**Keywords:** fibroblast attachment, enamel matrix derivatives, ethylenediaminetetraacetic acid, hyaluronic acid, scaling and root planing

## Abstract

Background: The wound-healing process incorporates a spectrum of periodontal therapeutic interventions that strive to restore the health and function of the periodontium. Fibroblasts play pivotal roles in tissue repair and regeneration. Extensive research has been focused on mechanical and chemical root surface modifications to enhance fibroblast adhesion, which is crucial for successful wound healing.

Purpose: This study aimed to assess the combined efficacy of mechanical and chemical root surface modifications in promoting fibroblast viability to root surfaces affected by periodontitis in comparison to chemical modifications alone.

Materials and methods: Root samples were collected from healthy individuals and those with advanced periodontitis. The specimens were prepared, and the experimental groups were categorized based on the type of surface modification with mechanical and/or chemical materials, including hyaluronic acid (HA), ethylenediaminetetraacetic acid (EDTA), enamel matrix derivatives (EMD), and EDTA/EMD. Fibroblasts were seeded onto previously treated root samples. Cell adhesion was assessed using a viability assay.

Results: Fibroblast viability was significantly higher on root surfaces treated with chemical agents than on those treated with mechanical and chemical modifications. Long-duration EDTA and short-duration EMD treatments were significantly effective in enhancing cell viability. EDTA/EMD surface treatments resulted in significantly higher cell viability in all groups compared to the periodontitis root surfaces.

Conclusion: EDTA, EMD, and their combined application can potentially ameliorate periodontitis-induced surface structural impairments. Mechanical surface debridement can significantly affect the effectiveness of EDTA and EMD root conditioning agents.

## Introduction

The wound-healing process is a highly intricate and dynamic biological response that goes beyond the mere repair of gingival injury and trauma. Instead, it encompasses a broader spectrum of conditions, including diverse periodontal therapeutic interventions such as non-surgical and surgical approaches. The primary objective of periodontal therapy is to re-establish the health and functionality of the entire periodontium [1], which comprises the gingiva, periodontal ligament, cementum, and alveolar bone.

A coordinated sequence of events initiates and guides the healing process upon periodontal therapeutic intervention. Fibroblast, a key player in this regenerative symphony, is critical in reconstructing damaged tissues [2]. These versatile fibroblasts, originating from various sources [3-6], converge at the wound site to execute reparative tasks. Fibroblasts undergo significant transformation at the wound site. They enter a proliferation phase, rapidly increasing in number to meet the demand for wound healing. These activated cells then synthesize and secrete essential components, including collagen fibers, fibronectin, elastin, and proteoglycans, forming the extracellular matrix (ECM) [7].

Extracellular matrix production is of utmost significance as it lays the groundwork for subsequent stages of tissue repair. This matrix is a thriving hub for establishing a vascular network and fostering an optimal environment for seamlessly integrating diverse cellular elements. Fibroblast proficiency in orchestrating collagen synthesis and remodeling significantly contributes to the overall success of wound healing. As healing progresses, fibroblasts diligently remodel the initially disorganized and less mature collagen fibers into more organized and functional arrangements [8]. This remodeling process is crucial for achieving appropriate tensile strength and structural integrity of the regenerated tissue. Fibroblasts actively participate in the secretion of various growth factors and cytokines that regulate and modulate healing. These bioactive molecules, including transforming growth factor-beta (TGF-β) [9] and Matrix Metalloproteinase (MMP) [10,11], have an important role in ECM remodeling and maturation.

Following an understanding of the indispensable role of fibroblasts in periodontal healing, extensive scientific inquiry has been undertaken to investigate diverse mechanical [12,13] and chemical root surface modifications [14-17]. The primary objective of these investigations was to reinstate the compromised root surface structure caused by the deleterious effects of periodontitis to a state conducive to encouraging fibroblast adhesion. The root surface is a pivotal determinant of fibroblast behavior [18] and can be effectively modified using mechanical, chemical, or combined approaches.

The use of hyaluronic acid (HA), ethylenediaminetetraacetic acid (EDTA), and enamel matrix derivatives (EMD) to treat root surfaces affected by periodontitis is based on their unique properties and potential benefits. Hyaluronic acid, known for its biocompatibility, aids in tissue healing and regeneration by supporting cell activity. As a chelating agent, EDTA removes mineralized deposits effectively, making the root surface easier to reach for subsequent treatments. Enamel matrix derivatives contain bioactive elements that stimulate periodontal regeneration. The rationale for utilizing these agents lies in their collective ability to address diverse challenges associated with periodontitis, offering a holistic approach to effective treatment.

The aim of this in vitro study is to test the hypothesis that mechanical root surface modifications by performing root planning procedures when used in conjunction with chemical root surface modifications, including hyaluronic acid, ethylenediaminetetraacetic acid, and enamel matrix derivatives, enhance fibroblast viability on root surfaces compromised by periodontitis. This is compared to cases where chemical root surface modifications are used without mechanical modifications. The null hypothesis is that there is no significant difference in fibroblast viability between root surfaces that undergo both mechanical and chemical modifications and those that undergo chemical modifications alone.

## Materials and methods

This study was approved by the local Scientific Committee of Umm Al Qura University (approval number UDZT270521). The consent form was obtained for the study purpose.

Teeth collection

Two distinct types of teeth were procured, namely, healthy and periodontitis-affected teeth. Healthy teeth were obtained from individuals referred to oral surgery clinics specifically for orthodontic extractions who displayed no clinical signs or symptoms of periodontitis. Periodontitis-affected teeth were collected from individuals with no significant medical history presented to a dental clinic with advanced-stage periodontitis (stages III and IV). The study included teeth with poor prognoses due to severe bone loss, extensive gingival inflammation, grade III mobility, and substantial calculus deposition. Healthy and periodontally affected extracted teeth were thoroughly examined to exclude root caries, root surface damage, or resorption. The teeth were preserved in saline solution-filled containers after removing the residual tissue with sterile moist gauze.

Sample pretreatment

In this study, 72 single-root teeth were collected. Scaling was performed on the teeth to remove all calculi from the root surface. The crowns of the teeth were separated from their roots using new disc burs. Each root was divided into two identically sized pieces to yield 144 equal pieces (n=144). The samples were thoroughly rinsed with distilled water and autoclaved. Finally, the prepared root samples were stored in saline containers throughout the data collection period.

Fibroblast cell isolation

Patients seeking dental treatment were referred to the periodontist clinic at Umm Al Qura Teaching Hospital for crown-lengthening procedures. The study participants were carefully selected to include healthy individuals without signs or symptoms of periodontal disease. Before the surgical intervention, the patients provided written informed consent and underwent a six-week plaque control regimen to ensure optimal oral health. During periodontal surgery, only keratinized soft-tissue samples that exhibited no evidence of inflammation or bleeding upon probing were collected. Soft-tissue biopsies were excised from the patient's oral cavity, carefully preserved in saline solution, and transported to the laboratory for fibroblast extraction.

Soft tissue specimens were thoroughly rinsed in the laboratory with phosphate-buffered saline (PBS). Gingival tissues were incubated with dispase (1 mg/mL; Sigma, USA) at 4°C for 12 h. Following incubation, the epithelial layer was carefully removed using a no. 15 blade and the connective tissue was sliced into small pieces. These tissue fragments were then cultured in a 25 mL flask containing an appropriate cell growth medium. The flask was placed in an incubator maintained at 37 °C with a humidified atmosphere containing 5% CO2. The complete growth medium used for cell culture was Dulbecco's Modified Eagle Medium (DMEM; Thermo Fisher Scientific, Waltham, MA, USA), supplemented with 10% fetal bovine serum (FBS; HyClone Thermo Fisher Scientific, Logan UT, USA), 100 U/mL penicillin, 100 mg/mL streptomycin, and 2.5 mg/mL amphotericin B (Thermo Fisher Scientific, Waltham, MA, USA).

Experimental Subjected Samples

The experimental design involved systematically categorizing the sample groups to investigate the effects of different chemical treatments on root surfaces with/without prior mechanical treatment by root planing methods operating Hu-Friedy curettes. This categorization resulted in four primary groups labeled: A (short chemical exposure without mechanical treatment), B (long chemical exposure without mechanical treatment), C (short chemical exposure with mechanical treatment), and D (long chemical exposure with mechanical treatment). Further subdivisions were established within each group, specifically subgroups one to six. Subgroups one and two served as control groups, representing healthy root surfaces and those affected by periodontitis, respectively. Subgroups three to six included root surface samples subjected to distinct chemical root conditioning agents. Subgroups three, four, five, and six were exposed to different chemical agents: HA Gel (Regedent AG, Zurich, Switzerland), EDTA 24% gel (Biodinâmica, Lisbon, Portugal), EMD (Straumann, Basel, Sweden), and combination of EDTA and EMD, respectively.

Specimens Surface Therapy

**Figure 1:**
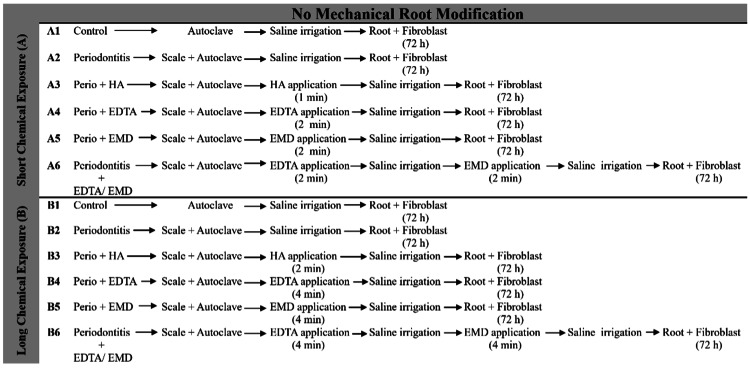
Experimental samples undergoing chemical treatment without concurrent mechanical modification A1= Untreated Healthy (Control), A2= Untreated Periodontitis, A3= Periodontitis treated with HA, A4= Periodontitis treated with EDTA, A5=Periodontitis treated with EMD, A6= Periodontitis treated with EDTA/EMD B1= Untreated Healthy (Control), B2= Untreated Periodontitis, B3= Periodontitis treated with HA, B4= Periodontitis treated with EDTA, B5= Periodontitis treated with EMD, B6= Periodontitis treated with EDTA/EMD HA: Hyaluronic Acid; EDTA: Ethylenediaminetetraacetic acid; EMD: Enamel Matrix Derivatives

**Figure 2:**
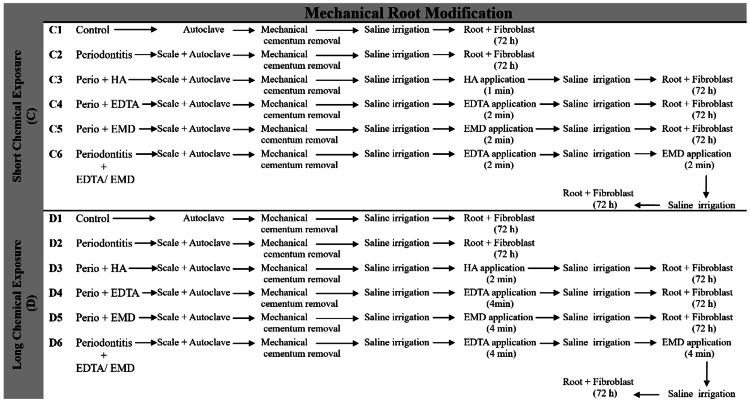
Experimental samples undergoing chemical treatment with concurrent mechanical modification. C1= Health treated with mechanical (Control), C2= Periodontitis treated with mechanical, C3= Periodontitis treated with mechanical/HA, C4= Periodontitis treated with mechanical/EDTA, C5= Periodontitis treated with mechanical/EMD, C6= Periodontitis treated with mechanical/EDTA/EMD D1= Health treated with mechanical (Control), D2= Periodontitis treated with mechanical, D3= Periodontitis treated with mechanical/HA, D4= Periodontitis treated with mechanical/EDTA, D5= Periodontitis treated with mechanical/EMD, D6= Periodontitis treated with mechanical/EDTA/EMD HA: Hyaluronic Acid; EDTA: Ethylenediaminetetraacetic acid; EMD: Enamel Matrix Derivatives

The experimental design involved the systematic categorization of samples into distinct groups. These groups comprised untreated healthy surfaces, denoted as A1 and B1 (representing the control groups without mechanical surface modification); mechanically treated healthy surfaces, designated as C1 and D1 (control groups with mechanical surface modification); untreated periodontitis surfaces, labeled as A2 and B2; mechanically treated periodontitis surfaces, identified as C2 and D2; surfaces subjected to chemical treatment, denoted as A3-A6 and B3-B6; and surfaces receiving combined mechanical and chemical treatment, categorized as C3-C6 and D3-D6. The experimental design, shown in Figures 1, 2, served as a well-structured framework for conducting this study.

Fibroblast Cell Adhesion to Root Surface

The root samples were carefully inoculated with a cell density of 2 × 10^4^ cells per well, and cell seeding was performed using 500 μL of complete growth medium. The inoculated root samples were incubated for 72 h.

Fibroblast Cells Viability Assessment

The root samples with adhered fibroblasts were transferred to 500 mL of DMEM containing 0.5 mg/mL of MTT (3-(4,5-dimethylthiazol-2-yl)-2,5-diphenyl-2H-tetrazolium bromide), followed by incubation at 37°C for 3 h. Following the incubation period, the samples were removed from the incubation medium, and the formazan crystals were dissolved by adding a solubilization solution composed of dimethyl sulfoxide (DMSO) and isopropanol in a 1:1 ratio. The solution was then poured into a 96-well plate at 100 mL per well. The cellular response was quantitatively evaluated by measuring each well's optical density (OD) at 570 nm using a spectrophotometer.

Statistical analysis

The cell viability within each group was summarized using the median and range, defined as the lowest to the greatest values. Statistical analysis was used to evaluate the impact of mechanical and chemical procedures on non-medicated root surfaces, both healthy and periodontally diseased. This study examined the impact of different durations of medication application (short vs. long) in both non-mechanical and mechanical groups. Each drug was compared independently. A comparative analysis examined the impact of non-mechanical and mechanical conditions under varying application durations for each medication individually. Subsequently, to assess the impact of the medications, cell viability in each medication group was classified based on whether mechanical treatment was performed and the duration of medication application. The viability of the cells in these groups was compared with that of the corresponding non-medicated groups, either healthy or periodontally diseased. The Mann-Whitney U test at a significance level of p <0.05 was used for the analytical comparisons.

## Results

**Figure 3:**
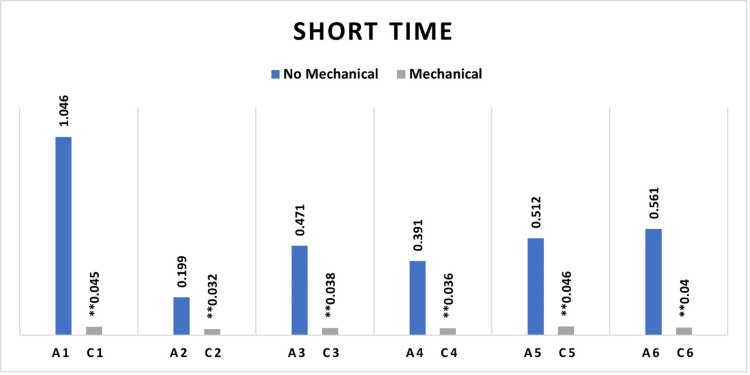
Median absorbance variations highlighting differences between healthy and periodontitis surface samples with short-duration chemical surface treatment, with and without prior mechanical surface instrumentation ** Significant statistical differences between the groups subjected to mechanical treatment and remaining untreated groups. A1= Untreated Healthy (Control), A2= Untreated Periodontitis, A3= Periodontitis treated with HA, A4= Periodontitis treated with EDTA, A5= Periodontitis treated with EMD, A6= Periodontitis treated with EDTA/EMD. C1= Health treated with mechanical (Control), C2= Periodontitis treated with mechanical, C3= Periodontitis treated with mechanical/HA, C4= Periodontitis treated with mechanical/EDTA, C5= Periodontitis treated with mechanical/EMD, C6= Periodontitis treated with mechanical/EDTA/EMD. HA: Hyaluronic acid; EDTA: Ethylenediaminetetraacetic acid; EMD: Enamel matrix derivatives.

**Figure 4:**
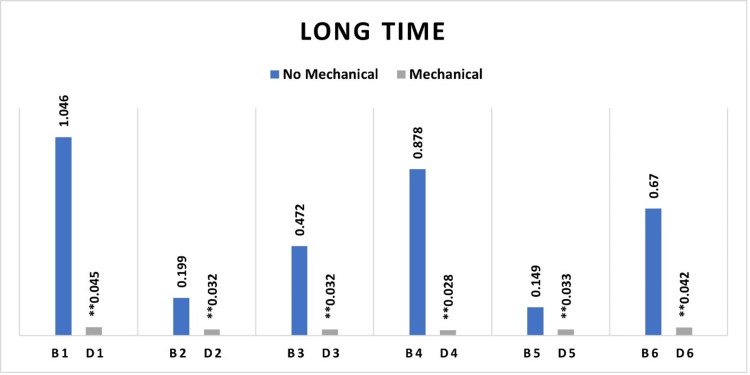
Median absorbance variations showing differences between healthy and periodontitis samples with long-duration chemical surface treatment, with and without prior mechanical surface instrumentation. ** Significant statistical differences between the groups subjected to mechanical treatment and remaining untreated groups. A significance level of p <0.05. B1= Untreated Healthy (Control), B2= Untreated Periodontitis, B3= Periodontitis treated with HA, B4= Periodontitis treated with EDTA, B5= Periodontitis treated with EMD, B6= Periodontitis treated with EDTA/EMD D1= Health treated with mechanical (Control), D2= Periodontitis treated with mechanical, D3= Periodontitis treated with mechanical/HA, D4= Periodontitis treated with mechanical/EDTA, D5= Periodontitis treated with mechanical/EMD, D6= Periodontitis treated with mechanical/EDTA/EMD HA: Hyaluronic acid; EDTA: Ethylenediaminetetraacetic acid; EMD: Enamel matrix derivatives.

Fibroblast cell viability was higher on root surfaces treated with chemical agents than on those treated with combined mechanical and chemical agents across all groups, including the control and experimental categories. The differences in cell viability reached a statistically significant threshold in all groups, including those with short and long chemical exposure durations (Figures 3, 4).

**Figure 5:**
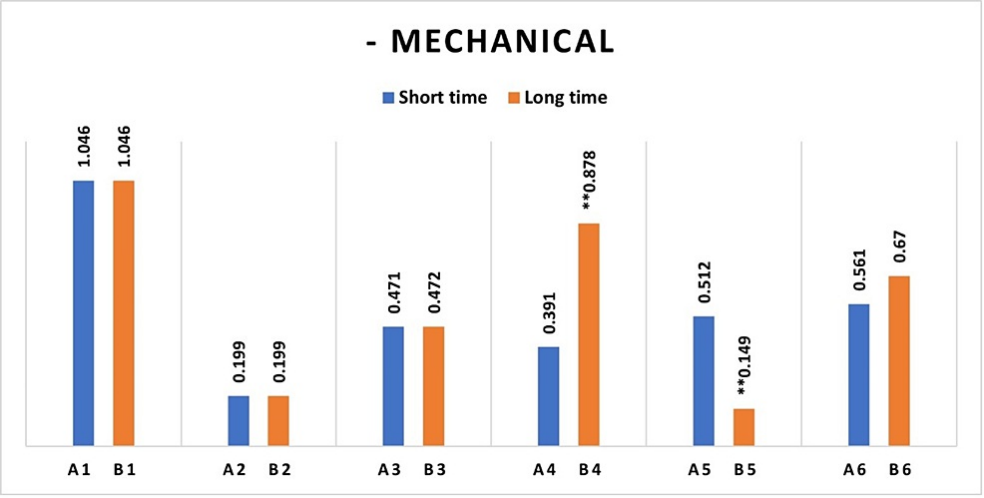
Median absorbance variations demonstrating disparities between samples subjected to short and long-duration chemical surface treatments without prior mechanical surface modification. ** Signifies statistically significant differences between groups exposed to chemicals with varying surface treatment durations A significance level of p <0.05. A1= Untreated Healthy (Control), A2= Untreated Periodontitis, A3= Periodontitis treated with HA, A4= Periodontitis treated with EDTA, A5=Periodontitis treated with EMD, A6= Periodontitis treated with EDTA/EMD. B1= Untreated Healthy (Control), B2= Untreated Periodontitis, B3= Periodontitis treated with HA, B4= Periodontitis treated with EDTA, B5= Periodontitis treated with EMD, B6= Periodontitis treated with EDTA/EMD. HA: Hyaluronic acid; EDTA: Ethylenediaminetetraacetic acid; EMD: Enamel matrix derivatives.

Cell viability was greater on root surfaces subjected to long EDTA chemical surface modifications compared to the group with short EDTA surface modifications, signifying a statistically significant difference between the two groups. Similarly, root surfaces treated with a short EMD chemical surface modification exhibited elevated fibroblast cell viability compared to those treated with extended EMD surface modification. The difference in cell viability between the two groups was significant (Figure 5).

When analyzing groups subjected to short-duration chemical exposure to HA (A3) and EDTA/EMD (A6) treatments without mechanical modification, a statistically significant difference in cell viability emerged compared to group A2; however, these differences did not reach significance compared to group A1. In the EDTA (A4) case, fibroblast cell viability demonstrated a statistically significant difference from that in groups A1 and A2 (Table 1).

**Table 1:** Median value comparative analysis of cell viability A1= Untreated Healthy (Control), A2= Untreated Periodontitis, A3= Periodontitis treated with HA, A4= Periodontitis treated with EDTA, A5=Periodontitis treated with EMD, A6= Periodontitis treated with EDTA/EMD. HA: Hyaluronic acid; EDTA: Ethylenediaminetetraacetic acid; EMD: Enamel matrix derivatives. Significance level: p <0.05, Sig/ S: significant, NS: Non Significant

Test (Sample + Material)	Control (Untreated Healthy)	P value	Sig
A3	A1	P=0.180	NS
0.471 (0.243 – 0.700)	1.046 (0.626 – 1.555)		
A4	A1	P=0.002	S
0.391 (0.359 – 0.403)	1.046 (0.626 – 1.555)		
A5	A1	P=0.180	NS
0.512 (0.148 – 1.004)	1.046 (0.626 – 1.555)		
A6	A1	P=0.180	NS
0.561 (0.331 – 0.869)	1.046 (0.626 – 1.555)		
Test (Sample + Material)	Periodontitis (Untreated Periodontitis)	P value	Sig
A3	A2	P=0.015	S
0.471 (0.243 – 0.700)	0.199 (0.136 – 0.253)		
A4	A2	P=0.002	S
0.391 (0.359 – 0.403)	0.199 (0.136 – 0.253)		
A5	A2	P=0.240	NS
0.512 (0.148 – 1.004)	0.199 (0.136 – 0.253)		
A6	A2	P=0.002	S
0.561 (0.331 – 0.869)	0.199 (0.136 – 0.253)		

The fibroblast cell viability exhibited statistically significant differences among groups subjected to EDTA (B4) and EDTA/EMD (B6) compared to group B2. These differences were not statistically significant compared to those in group B1 (Table 2).

**Table 2:** Median value comparative analysis of cell viability Experimental samples treated with long chemical treatment (B3-6) absorbance value compared to untreated healthy (B1) or untreated periodontitis-afflicted root (B2) samples. Significance level: p <0.05 B1= Untreated Healthy (Control), B2= Untreated Periodontitis, B3= Periodontitis treated with HA, B4= Periodontitis treated with EDTA, B5= Periodontitis treated with EMD, B6= Periodontitis treated with EDTA/EMD. HA: Hyaluronic acid; EDTA: Ethylenediaminetetraacetic acid; EMD: Enamel matrix derivatives; Sig/ S: Significant; NS: Non-Significant.

Test (Sample + Material)	Control (Untreated Healthy)	P value	Sig
B3	B1	P=0.002	S
0.472 (0.346 – 0.626)	1.046 (0.626 – 1.555)		
B4	B1	P=0.589	NS
0.878 (0.627 – 1.156)	1.046 (0.626 – 1.555)		
B5	B1	P=0.002	S
0.149 (0.131 – 0.160)	1.046 (0.626 – 1.555)		
B6	B1	P=0.180	NS
0.670 (0.282 – 1.206)	1.046 (0.626 – 1.555)		
Test (Sample + Material)	Periodontitis (Untreated Periodontitis)	P value	Sig
B3	B2	P=0.002	S
0.472 (0.346 – 0.626)	0.199 (0.136 – 0.253)		
B4	B2	P=0.002	S
0.878 (0.627 – 1.156)	0.199 (0.136 – 0.253)		
B5	B2	P=0.485	NS
0.149 (0.131 – 0.160)	0.199 (0.136 – 0.253)		
B6	B2	P=002	S
0.670 (0.282 – 1.206)	0.199 (0.136 – 0.253)		

Further variations in cell viability were observed in the context of mechanical modifications. Specifically, fibroblast cell viability was significantly higher in the groups subjected to EMD (C5) and EDTA/EMD (C6) than in group C2 (Table 3).

**Table 3:** Median value comparative analysis of cell viability Experimental samples subjected to short chemical treatment and mechanical surface alteration (C3-6) in contrast to healthy (C1) or periodontitis-afflicted roots (C2) with mechanical surface treatment. Significance level: p <0.05 C1= Health treated with mechanical (Control), C2= Periodontitis treated with mechanical, C3= Periodontitis treated with mechanical/HA, C4= Periodontitis treated with mechanical/EDTA, C5= Periodontitis treated with mechanical/EMD, C6= Periodontitis treated with mechanical/EDTA/EMD. HA: Hyaluronic acid; EDTA: Ethylenediaminetetraacetic acid; EMD: Enamel matrix derivatives; Sig/ S: Significant; NS: Non-Significant.

Test (Sample + Material)	Control (Mechanical Healthy)	P value		Sig
C3	C1	P=0.589		NS
0.038 (0.027 – 0.048)	0.045 (0.042 – 0.049)			
C4	C1	P=0.002		S
0.036 (0.033 – 0.039)	0.045 (0.042 – 0.049)			
C5	C1	P=1.00		NS
0.046 (0.036 – 0.057)	0.045 (0.042 – 0.049)			
C6	C1	P=0.093		NS
0.040 (0.034 – 0.044)	0.045 (0.042 – 0.049)			
Test (Sample + Material)	Periodontitis (Mechanical + Periodontitis)	P value	Sig	
C3	C2	P=0.699		NS
0.038 (0.027 – 0.048)	0.032 (0.027 – 0.033)			
C4	C2	P=0.026		S
0.036 (0.033 – 0.039)	0.032 (0.027 – 0.033)			
C5	C2	P=0.002		S
0.046 (0.036 – 0.057)	0.032 (0.027 – 0.033)			
C6	C2	P=0.002		S
0.040 (0.034 – 0.044)	0.032 (0.027 – 0.033)			

However, these differences were not statistically significant when compared with group C1. Fibroblast cell viability in the EDTA/EMD group (D6) was significantly higher compared to group D2. Nevertheless, the differences were not statistically significant compared to group D1 (Table 4).

**Table 4:** Median value comparative analysis of cell viability Experimental samples were subjected to long chemical treatment and mechanical surface alteration (D3-6) in contrast to healthy (D1) or periodontitis-afflicted roots (D2) with mechanical surface treatment. Significance level: p <0.05 D1= Health treated with mechanical (Control), D2= Periodontitis treated with mechanical, D3= Periodontitis treated with mechanical/HA, D4= Periodontitis treated with mechanical/EDTA, D5= Periodontitis treated with mechanical/EMD, D6= Periodontitis treated with mechanical/EDTA/EMD. HA: Hyaluronic acid; EDTA: Ethylenediaminetetraacetic acid; EMD: Enamel matrix derivatives; Sig/ S: Significant; NS: Non-Significant.

Test (Sample + Material)	Control (Mechanical Healthy)	P value	Sig
D3	D1	P=0.002	S
0.032 (0.028 – 0.036)	0.045 (0.042 – 0.049)		
D4	D1	P=0.002	S
0.028 (0.020 – 0.037)	0.045 (0.042 – 0.049)		
D5	D1	P=0.002	S
0.033 (0.024 – 0.041)	0.045 (0.042 – 0.049)		
D6	D1	P=1.00	NS
0.042 (0.031 – 0.051)	0.045 (0.042 – 0.049)		
Test (Sample + Material)	Periodontitis (Mechanical + Periodontitis)	P value	Sig
D3	D2	P=0.589	NS
0.032 (0.028 – 0.036)	0.032 (0.027 – 0.033)		
D4	D2	P=1.00	NS
0.028 (0.020 – 0.037)	0.032 (0.027 – 0.033)		
D5	D2	P=1.00	NS
0.033 (0.024 – 0.041)	0.032 (0.027 – 0.033)		
D6	D2	P=0.024	S
0.042 (0.031 – 0.051)	0.032 (0.027 – 0.033)		

Cell viability in all samples treated with mechanical and chemical agents in group C was not significantly different from that in group D (Table 5).

**Table 5:** Median value comparative analysis of cell viability Present quantification analysis of Cellular Viability adhered to root surfaces affected by periodontitis subsequent to mechanical surface treatment in conjunction with short (C) or long-duration (D) chemical surface alterations. Significance level: p <0.05 C3= Periodontitis treated with mechanical/HA, C4= Periodontitis treated with mechanical/EDTA, C5= Periodontitis treated with mechanical/EMD, C6= Periodontitis treated with mechanical/EDTA/EMD. D3= Periodontitis treated with mechanical/HA, D4= Periodontitis treated with mechanical/EDTA, D5= Periodontitis treated with mechanical/EMD, D6= Periodontitis treated with mechanical/EDTA/EMD. HA: Hyaluronic acid; EDTA: Ethylenediaminetetraacetic acid; EMD: Enamel matrix derivatives; NS: Non-Significant.

Test (Mechanical + Short-duration)	Test (Mechanical + Long-duration)	P value	Sig
C3	D3	P=0.818	NS
0.038 (0.027 – 0.048)	0.032 (0.028 – 0.036)		
C4	D4	P=0.180	NS
0.036 (0.033 – 0.039)	0.028 (0.020 – 0.037)		
C5	D5	P=0.180	NS
0.046 (0.036 – 0.057)	0.033 (0.024 – 0.041)		
C6	D6	P=1.00	NS
0.040 (0.034 – 0.044)	0.042 (0.031 – 0.051)		

## Discussion

The findings in this study highlight the critical role of the scaling of root surface alteration in fibroblast viability. Our investigation revealed that the root surfaces treated with chemical agents consistently exhibited higher fibroblast viability than those treated with a combination of mechanical and chemical modifications. This trend persisted across all study groups, including those with varying durations of treatment exposure. The significant disparities in cell viability observed among these groups indicated the profound influence of mechanical surface modifications on fibroblast attachment.

These results contrast with a study conducted by Qiu et al. 2020 [19], which investigated root surface topography from various mechanical instrumentation techniques. They concluded that the surface morphology altered by mechanical instruments enhanced cell adhesion behavior, surpassing the levels observed on untreated, healthy root surfaces. This discrepancy may be attributed to the differences in surface wettability. Qiu et al. noted that root surfaces treated with mechanical instruments exhibited increased wettability, which could contribute to the enhanced adhesion of gingival fibroblasts. However, it is necessary to acknowledge that both studies focused on root surface alterations and cellular responses. They may have involved different experimental contexts, variables, and methodologies, which could account for the differing outcomes.

Variations in fibroblast cell viability about the type and duration of chemical treatment highlight the importance of root surface treatment strategies for roots affected by periodontitis. The root surfaces subjected to long-duration EDTA chemical surface modification exhibited higher absorbance values than those subjected to short-duration EDTA treatment. This suggests that prolonged exposure to EDTA results in a rougher surface that promotes fibroblast adhesion [17]. This finding aligns with previous studies, indicating that EDTA, a chelating agent, can effectively remove calcium and phosphate ions from the root surface [20], rendering it more porous and conducive to fibroblast attachment [21].

Further trends were evident in root surfaces treated solely with short EMD chemical surface modifications, which exhibited increased fibroblast cell viability compared to surfaces subjected to extended EMD treatment. Parallel outcomes were achieved when short EMD chemical modifications were combined with surface mechanical alterations, and the total number of cells attached to the root surface was higher than that attached to the periodontitis surface modified with mechanical alterations. This emphasizes the potential advantages of exposure duration to specific chemical agents in facilitating fibroblast adhesion. EMD are derived from the porcine enamel matrix and promote new bone [22] and cementum [23] formation, making them a valuable component in periodontal regeneration.

The potential of EDTA and EMD to promote periodontal regeneration has been comprehensively evaluated. Research has revealed that applying EDTA or EMD on root surfaces holds promise for enhancing the clinical attachment level and upholding favorable clinical parameters in the vicinity of teeth affected by periodontitis [24,25].

Another outcome of this study was the substantial insight into the influence of EDTA/EMD treatment on fibroblast adhesion within the context of periodontal health and disease. When EDTA/EMD treatments were applied independently without concurrent mechanical surface modification, a significant enhancement in fibroblast adhesion was evident compared to the periodontitis-affected group. A similar sequence of enhanced adhesion in the study was observed when the EDTA/EMD treatments were combined with mechanical surface alterations [26,27]. The study highlights the effectiveness of these treatments for promoting fibroblast attachment when EDTA is combined with another chemical agent for periodontal tissue regeneration [17]. The results also showed that the disparities observed did not reach statistical significance compared to the healthy root group. The study suggests that EDTA/EMD treatment can modify the surface structure of periodontitis-affected roots.

Hyaluronic acid (HA) serves as a regulatory element in diverse biological processes [28] and has earned attention for its potential role in the treatment of periodontitis [29]. The effect of HA on P. gingivalis demonstrates its capacity to reduce inflammatory mediators and concurrently foster the migration and proliferation of fibroblast cells [30]. A distinctive observation from our study is HA increased fibroblast viability in the short application without supplementary mechanical modification, in contrast to surfaces altered by periodontitis. This observation implies a potential favorable influence of HA, particularly under concise application periods without additional mechanical interventions.

The main limitations of the study were that it had a limited sample size, the source of healthy teeth obtained from individuals referred for orthodontic extractions may not represent a random selection of healthy root surfaces, lack of long-term fibroblast behavior on the treated surface, and the study involved an in vitro setup, which does not fully replicate the complex in vivo conditions found in the oral cavity.

Future studies should expand the investigation of fibroblast adhesion to the root surface, employing diverse methodologies to comprehend cellular responses under varying conditions. Additionally, there is a need for experiments dedicated to scrutinizing the structural alterations that unfold on the root surface after applying chemical materials. Exploring long-term effects and the interplay between different chemical agents could significantly contribute to refining periodontal treatment strategies and advancing our understanding of the balance between chemical and mechanical factors in maintaining periodontal health.

## Conclusions

Despite the study's limitations, consistent findings reveal higher fibroblast cell viability on root surfaces treated solely with chemical agents than those receiving combined mechanical and chemical interventions. Statistically significant variations in cell viability were noted based on the duration of chemical exposure: EDTA 4 min yielded superior viability to EDTA 2 min, and EMD 2 min showed heightened viability compared to EMD 4 min. The group treated with HA for 1 min without mechanical intervention exhibited a notable increase in cell viability. Additionally, EDTA/EMD surface modification significantly amplified fibroblast cell viability in all groups except those with mechanical alteration and a combined 4-minute surface treatment of EDTA/EMD. The study suggests that the combined or singular application of mechanical and chemical treatments significantly influences therapeutic outcomes, with a preference for independent chemical root treatment.
